# Artificial Intelligence Model to Detect Real Contact Relationship between Mandibular Third Molars and Inferior Alveolar Nerve Based on Panoramic Radiographs

**DOI:** 10.3390/diagnostics11091664

**Published:** 2021-09-11

**Authors:** Tianer Zhu, Daqian Chen, Fuli Wu, Fudong Zhu, Haihua Zhu

**Affiliations:** 1 Stomatology Hospital, School of Stomatology, Zhejiang University School of Medicine, Clinical Research Center for Oral Disease of Zhejiang Province, Key Laboratory of Oral Biomedical Research of Zhejiang Province, Cancer Center of Zhejiang University, Hangzhou 310006, China; zhutianer@zju.edu.cn; 2 School of Computer Science and Technology, Zhejiang University of Technology, Hangzhou 310006, China; 2111912004@zjut.edu.cn (D.C.); fuliwu@zjut.edu.cn (F.W.)

**Keywords:** deep learning network, YOLOv4, mandibular third molar, inferior alveolar nerve, contact relationship, panoramic radiograph

## Abstract

This study aimed to develop a novel detection model for automatically assessing the real contact relationship between mandibular third molars (MM3s) and the inferior alveolar nerve (IAN) based on panoramic radiographs processed with deep learning networks, minimizing pseudo-contact interference and reducing the frequency of cone beam computed tomography (CBCT) use. A deep-learning network approach based on YOLOv4, named as MM3-IANnet, was applied to oral panoramic radiographs for the first time. The relationship between MM3s and the IAN in CBCT was considered the real contact relationship. Accuracy metrics were calculated to evaluate and compare the performance of the MM3–IANnet, dentists and a cooperative approach with dentists and the MM3–IANnet. Our results showed that in comparison with detection by dentists (AP = 76.45%) or the MM3–IANnet (AP = 83.02%), the cooperative dentist–MM3–IANnet approach yielded the highest average precision (AP = 88.06%). In conclusion, the MM3-IANnet detection model is an encouraging artificial intelligence approach that might assist dentists in detecting the real contact relationship between MM3s and IANs based on panoramic radiographs.

## 1. Introduction

The high impaction rate of mandibular third molars (MM3s) makes the extraction of third molars a common surgical procedure [1] that can result in multiple complications. Inferior alveolar nerve (IAN) injury is one of the most severe complications, resulting in hypoesthesia and numbness of the lower lip or chin [2]. The incidence of IAN injury ranges from 0.4~6% and IAN injury occurs most frequently when MM3s are closely related to the IAN [3,4]. There are various reasons for IAN injury after the extraction of MM3s, including direct trauma, indirect compression, or lack of bone cortex around the IAN [5]. The risk of IAN injury after tooth extraction increases when MM3s anatomically touch the IAN [6]. When the dental roots are in contact with the IAN, the bone cortex around the IAN may appear absent or discontinuous [7]. When the elevator is inserted into the periodontal ligament space of MM3s, a compressive load is generated in the apical region of the molar; the compressive load will act on the IAN during extraction and lead to IAN injury. Therefore, it is necessary to predict the contact relationship between MM3s and the IAN with radiographic examination before tooth extraction, which contributes to preoperatively predicting surgical difficulty and the possibility of complications [8,9], thereby developing a more minimally invasive extraction strategy and reducing the risk of IAN injury.

In radiographic examination, panoramic radiographs are most commonly used and aid dentists in determining the relationship between MM3s and the IAN canal because it can provide clinical dental image with short scan-time and low radiation dose [10]. However, panoramic radiographs have many shortcomings, such as anatomical noise, superimposition, and geometric distortion effect [11]. It can be difficult to distinguish the real contact relationship between MM3s and the IAN based on panoramic radiographs, especially when dental roots are located in the buccolingual direction of the IAN [12]. Pseudo-contact occurs frequently, which indicates that MM3s contact the IAN in panoramic radiographs, but this contact does not occur in cone beam computed tomography (CBCT). The visual detection of the relationship between MM3s and the IAN by dentists based on panoramic radiographs can thus be limited and unreliable [13]. Currently, the use of CBCT can reflect the three-dimensional structure of a tooth and the IAN to accurately distinguish the contact relationship between the dental roots and the IAN, which contributes to facilitating preoperative planning and reducing the risk of IAN injury [14]. However, CBCT is not used as a routine inspection method because it will significantly increase the patient costs and radiation dose [15,16], which doesn’t match the standard dose recommended in some countries [17]. Therefore, it is important to determine whether the real contact relationship can be precisely determined depending on panoramic radiographs, avoiding pseudo-contact issues and reducing the frequency of CBCT use.

Researchers have focused on the issue of pseudo-contact on panoramic radiographs. Studies have shown that when panoramic radiographs exhibit “darkening of the root”, “interruption of the radiopaque border of the mandibular canal”, and “inferior alveolar neural tube diversion” [18], the dental roots and IAN may display a close relationship, and the probability of IAN injury after extraction increases. However, the technique requires considerable training for dentists, and judgments with these methods are still not sufficiently accurate [19,20], especially for dental roots in the buccolingual direction. Overall, it is difficult but necessary to reliably detect the real relationship between MM3s and the IAN based on panoramic radiographs. Therefore, in this study, we use an artificial intelligence technique to aid in the diagnosis.

Deep learning networks have played an important role in medical image research, which can identify many complex image structures in modern medicine and have been used in various fields, such as multiple organ segmentation for the abdomen [21]. In stomatology, deep learning has also been applied in the detection of caries, periodontal disease, root development staging and other issues [22,23,24]. In terms of impacted teeth, few studies have focused on the relationship between impacted teeth and the IAN using deep learning. In previous studies, the researchers segmented and identified images of MM3s and IANs based on panoramic radiographs with a deep learning network called U-Net [25], but the accuracy of existing methods remains to be improved and the pseudo-contact of MM3s and the IAN in panoramic radiographs has not been mentioned. Within the limited scope of our knowledge, there has been no research on diagnostic models involving the real contact relationship between MM3s and the IAN with a deep learning network.

Therefore, in this study, we established a novel detection model for automatically assessing the real contact relationship between MM3s and the IAN based on panoramic radiographs and deep learning networks, named as MM3–IANnet. With this model, we sought to achieve two results: (1) minimizing interference from pseudo-contacts in panoramic radiographs, thereby reducing the frequency of CBCT use and (2) assisting dentists in more accurately identifying contact relationships, thereby estimating the risk of IAN injury more accurately before tooth extraction.

## 2. Materials and Methods

This study (ChiCTR2100044897) was approved by the Medical Ethics Committee of Stomatology Hospital, School of Stomatology, Zhejiang University School of Medicine, and was conducted in compliance with the ICH-GCP principles and the Declaration of Helsinki (2013).

### 2.1. Image Data Set

The study was conducted at Stomatology Hospital, School of Stomatology, Zhejiang University School of Medicine. The inclusion criteria for panoramic radiographs were as follows: (1) at least one mandibular third molar with fully developed dental roots must be present; (2) panoramic radiographs and CBCT scans less than 3 months apart from the panoramic radiographs must be available; and (3) patients must be older than 18 years old. Panoramic radiographs with buccolingual impacted of MM3s, incomplete panoramic radiographs, or panoramic radiographs of poor quality were not included in the study. All panoramic radiographs were acquired with a Dentsply Sirona (Bensheim, Germany) and an Orthophos XG 5OS Ceph.

**Figure 1:**
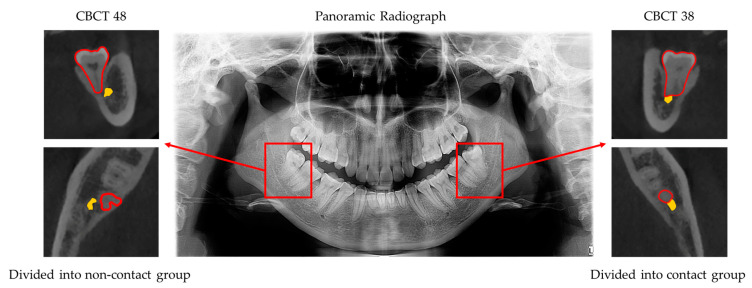
Panoramic view of a patient with corresponding CBCT results. Tooth position was recorded using the Federation Dentaire International system. Forty-eight showed that the dental roots were in contact with the IAN in the panoramic radiograph but not in contact in CBCT, so 48 was classified into the non-contact group. Thirty-eight showed the dental roots in contact with the IAN in both the panoramic radiograph and the CBCT result, so 38 was classified into the contact group.

All panoramic radiograph datasets were evaluated by three independent dentists who collected and categorized the results with kappa > 0.8. In total, 503 panoramic radiographs (915 MM3s) obtained between January 2016 and January 2021 were selected (age range of patients: 18 to 68 years old). The contact relationship between MM3s and the IAN canal in CBCT was considered the real contact relationship. Based on the real contact relationship between MM3s and the IAN in CBCT, these molars were divided into contact and non-contact groups. For individuals in the contact group, the dental roots of their molars were in contact with the IAN in CBCT, and vice versa for individuals in the non-contact group (Figure 1). The details of the two groups are shown in Table 1. The criteria for contact were as follows: (1) MM3s contacted with the mandibular canal with a defective white line and (2) MM3s penetrated the mandibular canal.

### 2.2. Deep Learning Network Construction and Training

The core mechanism of contact detection revolved around a deep learning network called YOLOv4, which had been verified to provide high accuracy and a fast analysis speed in the detection of ROIs [26]. We named our detection model as MM3–IANnet.

**Figure 2:**
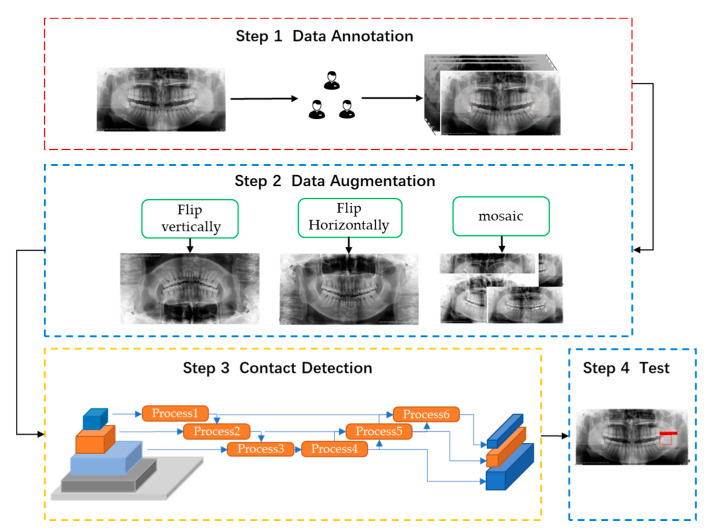
Model of MM3–IANnet system architecture. In step 3, process 1–process 3 was the upper sampling operation and process 4–process 6 was the lower sampling operation.

We used 80 percent of the images for training, 10 percent for validation and 10 percent for testing. The workflow of the model could be divided into four steps (Figure 2).

The first step was data annotation. In this step, all panoramic radiographs were resized to 1440 × 2976 pixels. When MM3s contacted the IAN canal in CBCT images, namely, MM3 were divided into contact group, the MM3s were labeled “touch” with the open-source software Labellmg. In total, 915 MM3s were included, with 549 for training, 183 for validation and 183 for network testing.

The second step was data augmentation. After labeling, we used three methods, namely, horizontal flipping, vertical flipping, and mosaicking, to enhance the data, which effectively expanded the number of datasets and improved training convergence.

The third step was touch detection. Images were input into YOLOv4. In this step, the workflow could be divided into three parts. The first part involved CSPDarkNet53, which was used to extract abundant feature information from the input images. Then SPP + PAN (space pyramid pooling module + path aggregation network) was used to generate feature pyramids. A feature pyramid could enhance the identification and detection of objects with different scales and sizes. YoloHead was used for the final test. The final output vector with class probability, object score, and bounding box information was the output.

The fourth step included inputting test data.

### 2.3. Diagnostic Performance Analysis

To compare the accuracy between the automated detection models MM3–IANnet and dentists, we randomly selected 188 MM3 as the testing dataset, and three dentists with 3 years of experience (Dentist 1, Dentist 2, and Dentist 3) and two dentists with 1 year of experience (Dentist 4 and Dentist 5) were asked to assess the dataset. Dentists were given background information about the study and the detection task. Furthermore, dentists were required to work cooperatively with the MM3–IANnet. We designed a voting experiment in which we set the weight of each dentist to 1 and the weight of the MM3–IANnet to 2. Firstly, the dentists and MM3-IANnet made independent judgements regarding the relationship between MM3s and the IAN based on the panoramic radiographs, and we then calculated the final test result according to the weighted results.

Based on the results for detection, the metrics were calculated to compare the performance of the deep learning network, the subjective assessments of dentists and the cooperative dentist–MM3–IANnet approach.

### 2.4. Statistical Analysis

Diagnostic accuracy was calculated using precision (TP/(TP + FP)), recall (TP/(TP + FN)), F1 score (2Precision*Recall/(Precision + Recall)) and average precision (AP = $\int_{0}^{1}p\left(r\right)dr$) (Table 2). A Chi-square test was used to compare the assessment results. Statistical analyses were performed with IBM SPSS Statistics 24.0, and the statistical level of significance was set to *p* < 0.05.

## 3. Results

### 3.1. Deep Learning Network Accuracy

After training, validation, and testing of 915 MM3s, the deep learning network YOLOv4, i.e., MM3-IANnet, yielded an average precision of 85.05%, a precision of 87.18%, a recall of 82.93% and a F1-score of 84.99%. Table 3 showed the detailed accuracy metrics of the new diagnosis model with YOLOv4 for detecting the real contact relationship between MM3s and the IAN based on panoramic radiographs.

### 3.2. Diagnostic Performance Analysis

Five dentists yielded an average precision of 76.45% ± 8.60%, a precision of 89.85% 6.81%, a recall of 83.00% ± 9.76% and a F1-score of 85.82% ± 5.06%. The mean average precision of dentists with 3 years of work experience (Dentist 1, Dentist 2, and Dentist 3) was 75.30% ± 11.02% (mean ± SD), and that of dentists with 1 year of work experience (Dentist 4 and Dentist 5) was 78.18% ± 6.57%. The intraclass correlation coefficient (ICC) of the five dentists was 0.302. Table 4 showed the detailed accuracy metrics for detections by dentists.

**Figure 3:**
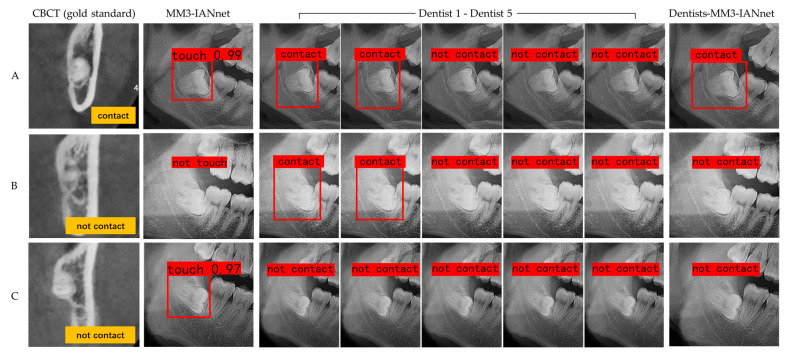
Output results of MM3–IANnet, dentists and cooperative dentist–MM3–IANnet approach. Three typical examples are presented. (**A**) According to the contact relationship in CBCT images (gold standard), the MM3 and IAN were divided into the contact group. The test result of MM3–IANnet was the contact. Two of the five dentists considered the case a contact, and the other three did not. The test result of dentist–MM3–IANnet (voting experiment) was a contact. (**B**) The MM3 and IAN were divided into the non-contact group. The test result of MM3–IANnet was the non-contact, and two of the five dentists considered the case a contact, while the other three did not. The test result of dentists–MM3–IANnet was the non-contact. (**C**) The MM3 and IAN were divided into the non-contact group. The test result of MM3-IANnet was a contact, five dentists considered the case non-contact, and the test result of dentist–MM3–IANnet was non-contact.

After testing of 188 MM3s, based on a comparison of diagnostic performance, MM3–IANnet yielded an average precision of 83.02%, a recall of 91.67% and a F1-score of 90.16%, which were higher than the mean average precision (76.45%), recall (83.00%), and F1-score (85.82%) of the five dentists. The cooperation between dentists and the MM3–IANnet, i.e., the voting experiment, yielded the highest average precision (88.06%), precision (93.88%), recall (92.00%), and F1-score (92.93%). The Chi-square test showed that the dentist–MM3–IANnet approach and MM3–IANnet were not statistically superior to the dentists-based assessment method (Figure 3 and Table 5; *p* > 0.05).

## 4. Discussion

The performance of a MM3–IANnet in detecting the real contact relationship between MM3s and the IAN based on panoramic radiographs was assessed in this paper, and the results were compared to those obtained by five dentists. We assumed the MM3–IANnet based on YOLOv4 yielded higher detection accuracy and reliability than the dentists, and the dentist–MM3–IANnet combination for detection was superior to the MM3–IANnet or dentists alone. Our findings partially support the original hypothesis that the MM3–IANnet yielded higher average precision, recall, and F1 score values than dentists, and the dentist–MM3–IANnet combination, i.e., the voting experiment, produced the highest average precision, precision, recall, and F1 score. However, statistical analysis showed the MM3-IANnet result and dentist–MM3–IANnet result were not statistically significant and superior to that of dentists.

In clinical practice, dentists often rely on experience to evaluate the contact relationship between MM3s and the IAN with the naked eye based on panoramic radiographs. Therefore, many studies have evaluated the predictive value of panoramic radiographs in assessing the relationship between MM3s and the IAN. Studies have shown that among the existing panoramic radiograph-based prediction methods, deflection of the root, narrowing of the root, dark and bifid apex of the root, and narrowing of the canal provide low predictive value, while the presence of a canal diversion, the interruption of the white line of the canal, and darkening of the root in panoramic radiographs could be routinely used to identify high-risk cases [19]. Still, the positive predictive value of these indicators was low and not sufficiently accurate [20]. Moreover, our results indicated that the average precision of the real contact relationship between MM3s and the IAN detected by dentists was only 76.45% ± 8.60%, which suggested that the accuracy was not high. Since there are limits to human assessment capabilities, artificial intelligence may be helpful in this field to identify the three-dimensional CBCT data in two-dimensional panoramic radiographs.

In our experiment, we applied YOLOv4 for detection with oral panoramic radiographs for the first time. YOLOv4, released in April 2020, is a new high-performance detection network that was developed based on the optimization of the previous convolutional neural network and has been applied in modern medicine [26,27]. YOLOv4 has a faster target detection speed and higher accuracy than other convolutional neural networks and has displayed excellent performance in many applications [28,29]. In YOLOv4, the only required input for the neural network to produce detection results is an image, and complex detection process can be avoided. Therefore, the detection speed for given targets is greatly improved. Moreover, YOLOv4 can avoid background errors, prevent false positives, and learn the general characteristics of target objects, thereby improving the detection accuracy. In our experiment, the core of model-based detection was YOLOv4, which performed rapid, real-time, lightweight, and accurate target identification under the premise of ensuring accuracy. Thus, this model could assist dentists in assessing the real contact relationship between MM3s and the IAN and improve the efficiency and accuracy of diagnoses. Therefore, this approach has excellent application potential in clinical practice.

Our study showed that with the application of MM3-IANnet, the predictive accuracy was increased, which in turn might decrease the frequency of CBCT use and IAN risk. The model of the real contact relationship detection between MM3s and the IAN based on panoramic radiographs was generally successful, as the total average precision of the MM3–IANnet was 85.05%. In the human–machine comparison experiment, the results showed that the mean precision of the five dentists was 89.85% ± 6.81%, indicating that the dentists had a certain ability to identify the contact relationship between the dental roots and the IAN from the panoramic radiographs, and their accuracy was acceptable when the dentists believed dental roots contacted the IAN; that is, the probability of dental roots and the IAN being in contact was high based on CBCT. However, the mean recall of dentists was 83.00% ± 9.76%, indicating that when the dental roots were judged by dentists to not be in contact with the IAN based on panoramic radiographs, there was still a high probability of contact based on CBCT. Therefore, the overall average precision of dentists was not high (76.45% ± 8.60%), suggesting that it was difficult for experienced dentists to accurately and comprehensively assess the real contact relationship between dental roots and the IAN in long-term clinical work. In the human–machine contrast experiment, although statistical analysis showed the MM3–IANnet result and dentist–MM3–IANnet result were not statistically significant and superior to that of dentists, the recall, F1 score, and average precision of the MM3–IANnet were numerically higher than those for dentists and the precision was close to that for dentists, indicating that MM3–IANnet at least possessed close ability to the dentists in detecting vague contact states of MM3s and IAN and might be superior to the dentists in accuracy.

In addition, the ICC of five dentists was 0.302 in our experiment, indicating that the five dentists’ judgments regarding the real contact relationship between MM3s and the IAN based on the same panoramic radiograph were in poor agreement. Some studies have shown that neither senior nor junior doctors could accurately assess the difficulty of wisdom tooth extraction based on panoramic radiographs, and even panoramic radiographs might hamper decision-making [9], which agreed with our results.

Our findings also suggested that in comparison to detection by dentists or MM3–IANnet independently, the approach in which YOLOv4 was combined with dentist assessment exhibited the highest average precision, precision, recall, and F1 score. The voting experiment was used to set the MM3–IANnet weights, thus enhancing MM3–IANnet accuracy while attenuating the effect of low detection consistency by the dentists. This finding indicated that the combination of the two methods yielded the most accurate results. Therefore, this approach could be clinically practical and has important application prospects.

However, this study had some limitations. The training dataset used in the model was not big enough, and not enough dentists were tested in the experiments. These factors might lead to deviations in the conclusions drawn, and further experimental verification is needed.

In addition to examining the real contact relationship between MM3s and the IAN based on panoramic radiographs, deep learning network can be used to mine more information from panoramic radiographs. The literature suggests that in anatomical studies of MM3s and the IAN, the dental roots are likely to be in close contact with IAN when the roots are buccal to the mandibular IAN, and the risk of nerve injury is high after tooth extraction [4,30]. Moreover, when MM3s are in the tooth germ state, the dental roots are far from the IAN, and the risk of IAN injury after tooth extraction is relatively low. If deep learning network can predict the contact relationship and anatomical position relationship between dental roots and the IAN when an MM3 is in the tooth germ stage, the result will provide important guidance in clinical practice.

## 5. Conclusions

In conclusion, this study applied a novel artificial intelligence detection model based on YOLOv4, named as MM3–IANnet, which might assist dentists in assessing the real contact relationship between MM3s and the IAN based on panoramic radiographs.

## Figures and Tables

**Table 1:** Results for Contact and Non-contact Group Categories.

Categories	Non-Contact Group	Contact Group
MM3 Number	530	328

**Table 2:** Confusion matrix.

		Actual Performance	
		1	0
Predicted Performance	1	True Positive (TP)	False Positive (FP)
	0	False Negative (FN)	True Negative (TN)

**Table 3:** Accuracy metrics of the MM3-IANnet for detecting real contact relationship.

Parameter	MM3-IANnet
Average precision	85.05%
Precision	87.18%
Recall	82.93%
F1-score	84.99%

**Table 4:** Detailed accuracy metrics of detecting ability of dentists.

Parameter	Dentist 1	Dentist 2	Dentist 3	Dentist 4	Dentist 5	Mean ± SD
Average precision	66.82%	71.33%	87.75%	73.53%	82.82%	76.45% ± 8.60%
Precision	95.45%	90.91%	97.22%	82.35%	83.33%	89.85% ± 6.81%
Recall	70.00%	76.92%	89.74%	84.00%	94.34%	83.00% ± 9.76%
F1-score	80.77%	83.33%	93.33%	83.17%	88.50%	85.82% ± 5.06%

**Table 5:** Performance comparison between the MM3–IANnet, dentists and cooperative dentist-MM3–IANnet approach.

Parameter	MM3-IANnet	Dentists (Mean)	Dentists-MM3-IANnet
Average precision	83.02%	76.45%	88.06%
Precision	88.71%	89.85%	93.88%
Recall	91.67%	83.00%	92.00%
F1-score	90.16%	85.82%	92.93%

## Data Availability

The data are not publicly available due to privacy. The data presented in this study are available on request from the corresponding author.
